# Supplementary material for: The Systems Biology Research Tool: evolvable open-source software
Source: BMC Syst Biol. 2008 Jun 29;2:55. doi: 10.1186/1752-0509-2-55 (PMC2446383; doi:10.1186/1752-0509-2-55)
Supplement: Additional file 1 — SBRT Archive. An archive of the current version of the Systems Biology Research Tool. [file 1752-0509-2-55-S1.zip › sbrt-1.4.0/doc/developers_guide/api/sbrt/kernel/util/class-use/UnmodifiableCollection.html]

Uses of Class sbrt.kernel.util.UnmodifiableCollection


|  |  |  |
| --- | --- | --- |
| **Overview** \| **Package** \| **Class** \| **Use** \| **Tree** \| **Deprecated** \| **Index** \| **Help** | |  |
| PREV   NEXT | **FRAMES**    **NO FRAMES**     **All Classes** |  |


---


## **Uses of Class sbrt.kernel.util.UnmodifiableCollection**

| Packages that use UnmodifiableCollection | |
| --- | --- |
| **sbrt.kernel.util** | Provides classes and interfaces for performing utility functions. |
| **sbrt.shell.io** | Provides classes and interfaces for reading and writing files. |

Uses of UnmodifiableCollection in sbrt.kernel.util

| Methods in sbrt.kernel.util that return UnmodifiableCollection | |
| --- | --- |
| `UnmodifiableCollection<V>` | `UnmodifiableMap.values()`             See Map.values(). |

Uses of UnmodifiableCollection in sbrt.shell.io

| Methods in sbrt.shell.io that return UnmodifiableCollection | |
| --- | --- |
| `UnmodifiableCollection<java.lang.Character>` | `TextInputFile.getCommentChars()`             Returns the commment characters used in this file. |

---


|  |  |  |
| --- | --- | --- |
| **Overview** \| **Package** \| **Class** \| **Use** \| **Tree** \| **Deprecated** \| **Index** \| **Help** | |  |
| PREV   NEXT | **FRAMES**    **NO FRAMES**     **All Classes** |  |


---
